# Revealing the potential of necroptosis-related genes in prognosis, immune characteristics, and treatment strategies for head and neck squamous cell carcinoma

**DOI:** 10.1038/s41598-023-47096-7

**Published:** 2023-11-21

**Authors:** Junhua Cao, Congxiao Wu, Zhaofeng Han, Zheng Liu, Zheng Yang, Minge Ren, Ximei Wang

**Affiliations:** 1 Plastic Surgery of the First Affiliated Hospital of Zhengzhou University, 1 East Road, JianShe, Erqi District, Zhengzhou City, 450052 Henan China; 2 Department of Plastic and Cosmetic Surgery, Nanfang Hospital, Southern Medical University, Guangdong, China

**Keywords:** Cancer, Cancer genomics, Head and neck cancer, Tumour immunology

## Abstract

Necroptosis is a recently discovered apoptotic mechanism that has been linked to tumor formation, prognosis, and treatment response. However, the relationship between the TME and NRGs remains unclear. In this study, we analyzed the expression patterns of NRGs in 769 HNSCC cases from two distinct data sets. Our findings revealed distinct genetic groups and a correlation between patient clinical features, prognosis, TME cell infiltration characteristics, and NRG alterations. We then developed an NRG model to predict OS and confirmed its accuracy in predicting OS in HNSCC patients. Moreover, we have devised a precise nomogram that enhances the clinical utility of the NRG model substantially. The low-risk group had a better OS, and they were associated with immune suppression, more mutated genes, and higher TIDE scores. The risk score also had a significant correlation with the CSC index and susceptibility to anti-tumor agents. Our study provides insights into how NRGs affect prognosis, clinically significant features, TME, and immunotherapy response in HNSCC. With a better knowledge of NRGs in HNSCC, we could assess the prognosis and develop immunotherapy regimens that are more successful at opening up new doors.

## Introduction

Head and neck squamous cell carcinoma (HNSCC) represents a major global public health challenge, standing as the sixth most prevalent malignancy on a worldwide scale^1^. Predictions suggest that there will be a 30% increase in the risk of HNSCC by 2030, with an estimated 1.08 million new diagnoses per year^2^. Notably, HNSCC survivors have the second-highest risk of suicide among cancer survivors, after those with pancreatic cancer. This risk is often linked to mental suffering and poor quality of life^3^. Despite comprehensive treatment approaches combining chemotherapy and surgery, the median overall survival (OS) of HNSCC patients remains less than 1 year, indicating a significant need for reliable biomarkers in diagnosing, prognosing, and treating HNSCC^4^. These alarming statistics highlight the pressing need for biomarker identification in HNSCC to improve patient outcomes^5^. Furthermore, biomarkers can guide personalized treatment approaches, such as targeted therapies, that may maximize treatment efficacy while minimizing toxicity. It is essential that future research prioritize the identification and validation of biomarkers to improve HNSCC patient outcomes.

Necroptosis, a type of regulated cell death, is mediated by MLKL, RIP1, and RIP3 and has been implicated in the pathogenesis of several diseases, including cancer, infectious diseases, and Parkinson’s^6–8^. While necroptosis can promote tumor growth and T cell death, it has also been linked to the antitumor immune response^6^. Recent studies have investigated the association between necroptosis-related genes (NRGs) and the prognosis and immune infiltration of several cancers, such as soft tissue sarcoma and breast cancer^9^. Scores related to necroptosis have been shown to predict a better prognosis and increased immune infiltration in these cancers^10^. Necroptosis is also a crucial cellular reaction that regulates the beginning, development, and spread of cancer. Researchers have shown that indicators for the prognosis of specific cancers and diseases may include necroptosis regulators^11,12^. Moreover, NRGs have been integrated into models to predict overall survival in patients with HNSCC^13^. While necroptosis regulators have the potential to serve as prognostic indicators for certain cancers and diseases, the molecular mechanisms underlying their effects on HNSCC outcomes remain unclear. The release of CXCL5 from necrotic lesions in pancreatic cancer has been linked to tumor cell invasion and migration^14^. Future studies should aim to elucidate the cellular and molecular mechanisms underlying the impact of necroptosis regulators on HNSCC pathogenesis, prognosis, and antitumor immune response to facilitate the development of personalized treatment strategies.

The utilization of immunological checkpoint blocking has demonstrated exceptional clinical efficacy in a small number of patients, with extended therapeutic responses. However, a significant portion of patients exhibited limited or no therapeutic benefit and failed to meet clinical parameters^15^*.* The oncogenic transformation of cancer cells alters their biological behavior by inducing immunological tolerance, impeding angiogenesis and apoptosis, and prompting hypoxia and proliferation via targeting certain tumor microenvironment (TME) components, both directly and indirectly. Notably, Ting Hu et al. discovered a substantial association between the necroptosis risk score for breast cancer and immune infiltration^16^*.* Consequently, it is projected that a comprehensive analysis of the complexities and variances within the TME landscape will enable the identification of differing tumor immune phenotypes, thus enhancing the ability to forecast and guide immunotherapeutic responses. The discovery of accurate biomarkers capable of assessing a patient's response to immunotherapy will assist in identifying novel therapeutic targets^17,18^*.*

With the swift progression of scientific research, coupled with the availability of TCGA and GEO databases, comprehensive analyses of the metabolome, transcriptome, genome, and proteome have become possible. Such analyses enable the identification of biomarkers and mechanisms governing carcinogenesis for therapeutic interventions and the prognosis. In the context of our research, we explored the expression patterns, prognostic significance, and potential regulatory networks of NRGs in HNSCC. Our findings are anticipated to contribute to our understanding of the molecular mechanisms and prognostic biomarkers in HNSCC.

## Materials and methods

### Preparation of data

In our investigation of gene expression and its relevance to important prognostic and clinical factors in HNSCC, we utilized data obtained from both the TCGA-HNSCC and GEO-GSE65858 databases^19^. The TCGA and GEO databases offer a rich resource of genomic, transcriptomic, and clinical data on various cancer types, allowing for in-depth analyses of disease mechanisms, biomarker discovery, and therapeutic targeting. In order to achieve compatibility of TPM values in HNSCC with those in microarray datasets, conversion to FPKM values was undertaken. The combination of two datasets was then performed, with batch effects subsequently corrected through the implementation of R packages "limma" and "sva"^20,21^. From a total of 796 HNSCC patients examined in this study, those lacking information regarding OS were excluded.

### Consensus clustering analysis

For the identification of NRG expression subgroups, a set of 67 NRGs was culled from published sources^22–24^. We used a ssGSEA algorithm to assess differences in necroptosis scores in early and advanced tumors. Using the R package "ConsensusClusterPlus" with kmeans clustering and euclidean distance, unsupervised clustering analysis was performed to classify patients into distinct molecular subtypes based on NRG expression^25^. The classification was based on the following criteria: (1) a smooth and steady rise in the cumulative distribution function curve; (2) avoidance of small sample sizes within any group; and (3) a decrease in inter-group correlations coupled with increased intra-group correlations.

### Clinical features, prognosis, and molecular subtype correlation

To evaluate the clinical utility of the NRG clusters recognized by consensus clustering, we investigated the interplay between genetic subtypes, clinical attributes, and prognostic outcomes. The analysis divulged insights into potential biomarkers, which could be vital for optimal medical interventions in clinical practice. In addition, to elucidate OS disparities among various subtypes, we employed Kaplan–Meier curves, a statistical tool that offers a graphical depiction of survival probability over time. The utilization of the "survival" and "survminer" R packages enabled us to generate accurate and reliable curves to provide a nuanced understanding of the disease progression trajectories. Moreover, we utilized PCA analysis to investigate the distribution of different NRG clusters within the HNSCC population. To explore variations in NRG biological processes, GSVA was employed^26^.

### Connection between molecular subtypes and TME

Moreover, we harnessed the capabilities of the CIBERSORT algorithm to compute the scores representing the abundance of 22 distinct immune cell types in each sample of HNSCC^27^. The CIBERSORT algorithm is a powerful computational tool used to deconvolve bulk gene expression data and estimate the relative abundances of different cell types within complex tissue samples. This analysis allowed for a comprehensive understanding of the immune cell infiltration within the TME. To investigate the levels of immune cell infiltration, we utilized the ssGSEA algorithm^28^. Unlike traditional GSEA, which compares the distribution of gene sets between two groups, ssGSEA computes a single enrichment score for each sample based on the relative expression of genes within the gene set. This technique enabled us to accurately assess the degree of immune cell infiltration in individual samples and identify differentially regulated immune pathways.

### Enrichment analysis

Using the "limma" package in R, we successfully recognized differentially expressed genes (DEGs) in different NRG clusters (*p* value < 0.05 and |logFC|> 0.585). The investigation of these DEGs was central to unraveling the underlying molecular mechanisms in the necroptosis cluster, a critical cell death pathway that plays a vital role in several diseases. To better understand the functional implications of these DEGs, we utilized the "clusterprofiler" package in R, a powerful tool for performing functional enrichment studies. Finally, based on these DEGs, unsupervised cluster analysis was again performed, and the Kaplan–Meier analysis was repeated.

### Construction of prognostic model

To identify DEGs that correlate with the OS of HNSCC patients, we conducted a univariate Cox analysis on the DEGs. Further, an unsupervised clustering method was employed, leveraging the prognostic DEGs, to stratify HNSCC patients into distinct subtypes, such as gene clusters A and B. We then randomly divided the HNSCC patients into training and testing sets at a 7:3 ratio to develop a prognostic NRG model. To reduce the chances of over-fitting and enhance model robustness, we used LASSO Cox analysis. The LASSO method is particularly useful for selecting a subset of relevant predictors among a larger set of potential predictors. The Cox regression model, on the other hand, is a popular survival analysis tool that can be used to investigate the relationship between multiple predictors and the time-to-event outcome. The present study employed a risk assessment method that involved the computation of risk scores utilizing the following mathematical expression: risk score = Σ(expi * coefi). The variables expi and coefi referred to the expression value and risk coefficient of each gene, respectively. The distribution of patients into various risk categories was based on the median value of the computed risk scores. Relationships between NRG clusters, gene clusters, risk groups, and survival status are represented in Sankey diagrams, and differences in risk scores in different clusters were examined. Subsequently, the accuracy of the developed prognostic model was assessed via the application of Kaplan–Meier analysis and ROC curves, which were utilized to analyze the distinct risk groups.

### Development of nomogram

To develop a comprehensive and personalized prediction model for HNSCC survival outcomes, we integrated the risk scores derived from the NRGs model and several clinical features using a scoring system called a nomogram. In this system, each variable is assigned a score, which is then summed to obtain the total score^29^. The nomogram provides an intuitive way to estimate the probability of survival for an individual patient based on their unique clinical features and molecular risk profile. To assess the accuracy of the model, we employed a nomogram calibration plot, which plots the predicted values against the actual survival outcomes at different time points, including 1-, 3-, and 5-years. The plot provides a visual representation of the performance of the nomogram and enables clinicians to adjust and refine the model to optimize its accuracy.

### Evaluation of immune status

In order to gain a comprehensive understanding of the immune infiltration in HNSCC, we utilized the CIBERSORT algorithm to measure the abundance of 22 infiltrating immune cells. Subsequently, we investigated the potential relationships between the levels of these immune cells and the risk score and genes in the prognostic NRGs model. Moreover, we investigated the expression levels of immune checkpoint genes and the TME score in the subtypes (clusters A and B).

### Analysis of mutations, immunotherapy responses and drugs

To gain insights into the molecular basis of HNSCC progression and prognosis, we investigated the somatic mutations in the identified risk groups and explored potential correlations with the cancer stem cell (CSC) index and tumor immune dysfunction and exclusion (TIDE), which are key indicators of tumor immune dysfunction and exclusion. Moreover, we computed the IC50 values of the commonly used chemotherapeutic drugs for HNSCC using the R package "pRRophetic". This approach allowed us to estimate the drug sensitivities of individual patients and identify potential therapeutic strategies that may be effective for specific subtypes of HNSCC.

### Cell culture

The human oral epithelial cell carcinoma (SCC25) and human oral epithelial cell line (WSU-HN30) were procured from iCell Bioscience Inc, Shanghai, China (iCell-h361) and Shanghai Zephyr Biotechnology Co., Shanghai, China (ZYH60432), respectively. These cells were cultured in DMEM medium (Gibco, New York, NY, United States) supplemented with 10% FBS (Gibco) serum, and maintained at 37 °C with 5% CO2 in a humidified incubator. Penicillin–streptomycin (100 Units/ml each, MedChemExpress, Shanghai, China) were added to the medium. Medium replacement was carried out every 2 days, while cells beyond passage 10 were not considered for experimental purposes.

### RT-PCR analysis

Total RNA was extracted from the hippocampus using Trizol® reagent and its quality and concentration were evaluated using 1% agarose gel electrophoresis and a Q3000 micro-volume spectrophotometer, respectively. Subsequently, the extracted RNA was reverse transcribed into cDNA using the RevertAid First Strand cDNA Synthesis Kit. The primer sequences utilized in this study, which were designed by Sangon Biotech Co., Ltd. based on published mRNA sequences in the NCBI, are presented in Table 1. The Multicolor Real-time PCR Detection System was employed for the qRT-PCR reaction using SYBR ® Green PCR Master Mix in a final volume of 25 L. The thermal cycling conditions comprised preincubation at 94 °C for 3 min, denaturation at 94 °C for 30 s, annealing at 55 °C for 30 s, and elongation at 72 °C for 30 s for a total of 40 cycles. Signal normalization was performed with respect to HPRT1 and the 2^−ΔΔCt^ method was used to calculate the relative mRNA expression in each sample.

**Table 1:** The primer sequences used for PCR amplifcation.

Gene	–	Sequences
THBS1	Froward	CCAATGCGACTTACCACTGC
	Reverse	CGGTCTCCCACATCATCTCTG
EFNB2	Froward	GGAGGAGACACAGGAAGCAC
	Reverse	CGTAGTGAGGGCAGAAGACG
P4HA1	Froward	GGGGTTGCTGTGGATTACCT
	Reverse	CGAGGCTTGTCCCATTCATC
F2RL1	Froward	TGTCCTCACTGGAAAACTGACC
	Reverse	GCAAACCCACCACAAACACAA
GAPDH	Froward	GCACCGTCAAGGCTGAGAAC
	Reverse	TGGTGAAGACGCCAGTGGA

### Validation of prognostic signature

In this study, we employed the HPA database to analyze the protein expression levels of NRGs in both normal and tumor tissues^30^. Our analysis aimed to provide insights into the expression patterns and localization of these genes in normal and tumor tissues. Through the use of the HPA database, we were able to conduct a comprehensive analysis of protein expression levels that can serve as a valuable resource for future research in this field.

### Western blotting analysis

RIPA lysate buffer containing a protease inhibitor and a phosphatase inhibitor was employed to treat SCC25/WSU-HN30 cells. Using a BCA kit, protein concentrations in supernatants were measured. After loading proteins (20 μl per lane) and markers (5 μl per lane) into SDS-PAGE gel wells, electrophoresis was used to separate the proteins by molecular weight (8–10% SDS-PAGE separation gel and a 5% SDS-PAGE concentration gel). Next, proteins were transferred using a wet transfer technique on polyvinylidene fluoride membranes (PVDF, Solarbio). PVDF membranes that were bound to proteins were blocked by submersion in 5% (w/v) bovine serum albumin from Solarbio, and then membranes were probed with antibodies: anti-Thrombospondin 1 Polyclonal Antibody (THBS1), YT6293; anti-EFNB2 Polyclonal Antibody (EFNB2), YN0096, anti-P4HA1 rabbit pAb (P4HA1), YT7464; anti-PAR2 Polyclonal Antibody (F2RL1), YN2681 as dilutions of 1:2000, GAPDH, YM3029, as dilutions of 1:8000, followed by incubation at 4 °C overnight. Next, membranes were incubated with secondary antibodies (HRP) at room temperature for 1 h. An enhanced ECL, BL523B reagent, is added to the film, developed after exposure with blue film, fixed, and scanned by a scanner for analysis.

### Statistical analyses

All statistical analyses were performed using R software version 4.2.0. The Wilcoxon test was employed to investigate the expressions across the various groups. Unless specifically stated, a significance level of *p* < 0.05 was deemed statistically significant.

## Results

### Necroptosis-related Genes in HNSCC

**Figure 1:**
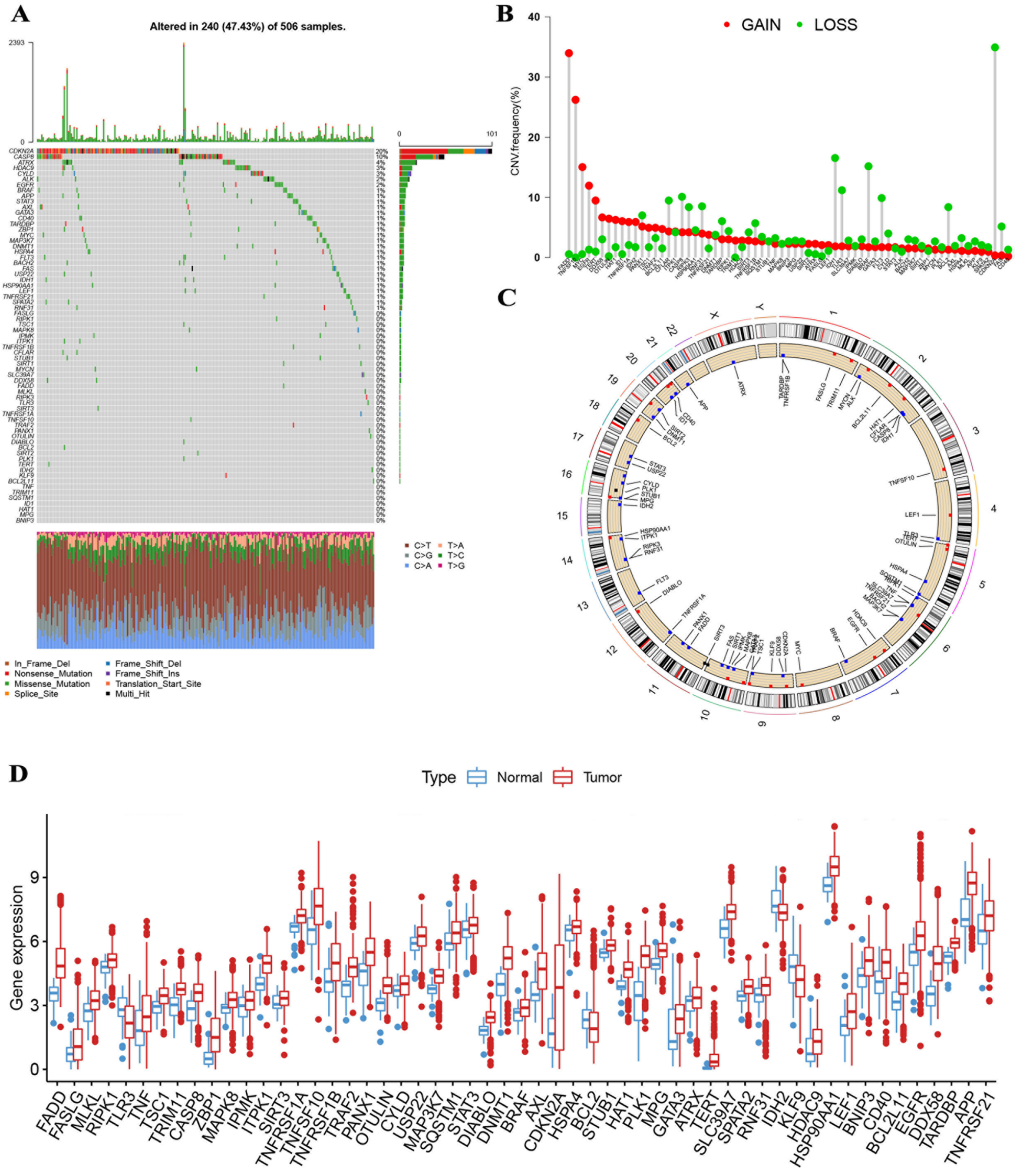
(**A**) The highest mutation frequency is CDKN2A (20%) among them, followed by CASP8, while 38 NRGs (BNIP3, MPG, HAT1 et al.) were almost mutation-free. (**B**) FADD, TNFSF10, MYC, EGFR, and TEGR with general CNV increases, and CDKN2A, IDH1, BRAF, TLR3, and FLT3 exhibiting CNV decreases. (**C**) The positions of the CNV alterations on chromosomes for these NRGs. (**D**) NRGs with CNV deficiencies, such as CDKN2A, IDH1, BRAF, TLR3, and FLT3, demonstrated lower expression levels relative to those in normal HNSCC samples; however, those with CNV gains, including FADD and TNFSF10, exhibited significantly elevated expression levels in HNSCC samples.

We explored 67 NRGs in our research (Tab S1). Necroptosis scores did not differ significantly between early and advanced tumors (Fig S1). The incidence of somatic mutation frequency was relatively high in the HNSCC cohort when we analyzed these 67 NRGs (Fig. 1A). The highest mutation frequency is CDKN2A (20%) among them, followed by CASP8, while 38 NRGs (BNIP3, MPG, HAT1 et al.) were almost mutation-free. In this study, we aimed to examine somatic copy number variations (CNVs) in a cohort of 67 NRGs. We detected prevalent CNV alterations in several NRGs, including FADD, TNFSF10, MYC, EGFR, and TEGR with general CNV increases, and CDKN2A, IDH1, BRAF, TLR3, and FLT3 exhibiting CNV decreases (Fig. 1B). The distribution of CNV changes on chromosomes among the NRGs is graphically depicted in Fig. 1C. We observed that NRGs with CNV deficiencies, such as CDKN2A, IDH1, BRAF, TLR3, and FLT3, demonstrated lower expression levels relative to those in normal HNSCC samples; however, those with CNV gains, including FADD and TNFSF10, exhibited significantly elevated expression levels in HNSCC samples (Fig. 1D). These discoveries indicate that CNV may play a regulatory role in the expression of NRGs. Notably, we did not observe any difference in the frequency of CNV gains or losses in NRGs between tumor and normal samples, except for GSDMB, which exhibited a downregulation of mRNA expression. Therefore, whereas CNV could interpret numerous changes in NRG expression, it does not represent the only factor participating in mRNA expression regulation^31^. DNA methylation and transcription factors could modulate gene expression as well^32,33^. Our analysis indicated that the potential function of NRGs in HNSCC oncogenesis showed a difference in both the expression levels of NRGs and the genetic profiles.

### Classification of necroptosis-related subtypes

**Figure 2:**
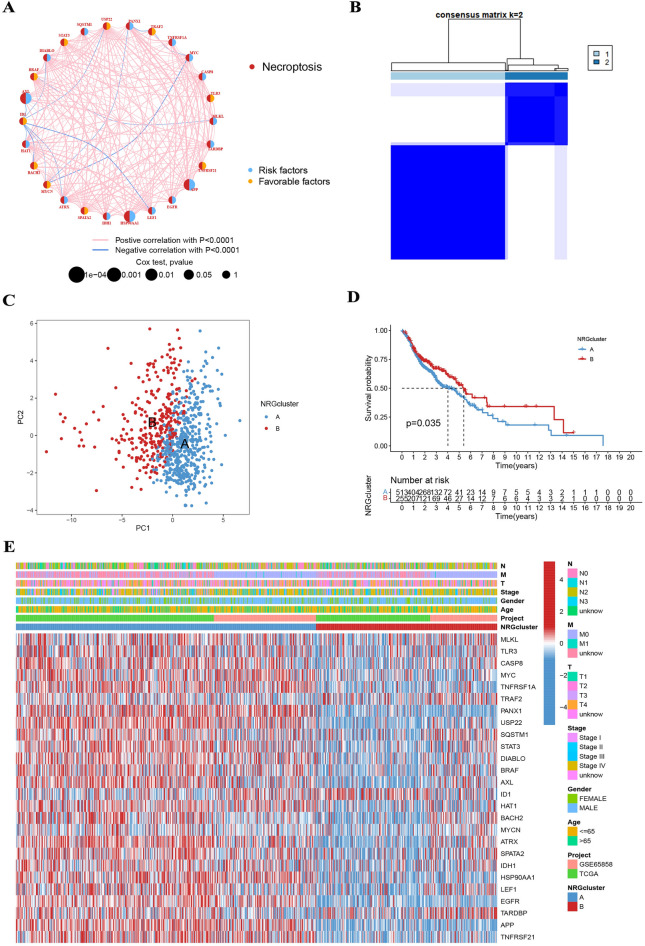
(**A**) The general set of NRG interactions, regulator connectivity and prognostic value was determined in the necrosis network. (**B**) Consensus matrix heatmap defining two NRG clusters (k = 2). (**C**) PCA analysis indicated that the transcriptional profiles of necroptosis disease were clearly different between different subtypes. (**D**) Kaplan–Meier curves show that NRG cluster B patients have higher OS. (**E**) Differences in clinical features and NRG expression levels between the two NRG subtypes.

We chose 796 patients from two eligible HNSCC cohorts (TCGA and GSE65858) to integrate for further analysis and to investigate the expression pattern of NRG. We screened for 26 NRGs significantly associated with prognosis using univariate Cox analysis. The general set of 26 NRG interactions, regulator connectivity, and prognostic value were determined in the necrosis network (Fig. 2A). To probe the expression patterns of NRGs in HNSCC, we grouped the patients with HNSCC on the basis of the expression profiles of the 26 NRGs by using a consensus clustering algorithm. Based on the above results, we classified the cohort into NRG clusters A and B (Fig. 2B). In PCA analysis, the transcriptional profiles of necroptosis disease were clearly different between the two subtypes (Fig. 2C). Kaplan–Meier curves show that NRG cluster B patients have a higher OS (*p* = 0.035; Fig. 2D). Moreover, we finally learned that NRG expression and clinicopathological features were significantly different, as demonstrated by comparing clinical features of different HNSCC subtypes (Fig. 2E).

### Assessment of TME

**Figure 3:**
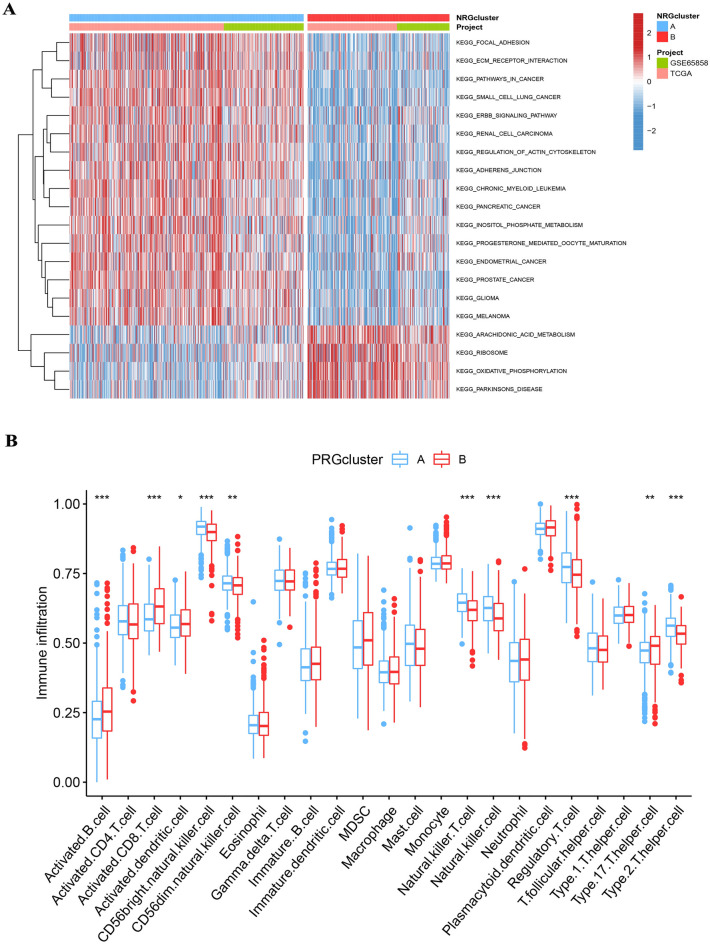
(**A**) NRG cluster A was remarkably abundant in focal adhesion, ECM receptor interaction, pathways in cancer, and small cell lung cancer, while NRG cluster B was significantly enriched in arachidonic acid metabolism, ribosome, oxidative phosphorylation, and Parkinson’s disease. (**B**) The two subtypes differed significantly in the infiltration of certain immune cells.

GSVA analysis indicated that NRG cluster A was remarkably abundant in focal adhesion, ECM receptor interaction, pathways in cancer, and small cell lung cancer, while NRG cluster B was significantly enriched in arachidonic acid metabolism, ribosome, oxidative phosphorylation, and parkinson’s disease (Fig. 3A and Tab S2). We use the CIBERSORT algorithm to explore the NRGs in the TME of HNSCC, assessing the correlations between the 22 immune cell subsets and two subtypes of every HNSCC sample (Fig. 3B).

### Classification of gene clusters

**Figure 4:**
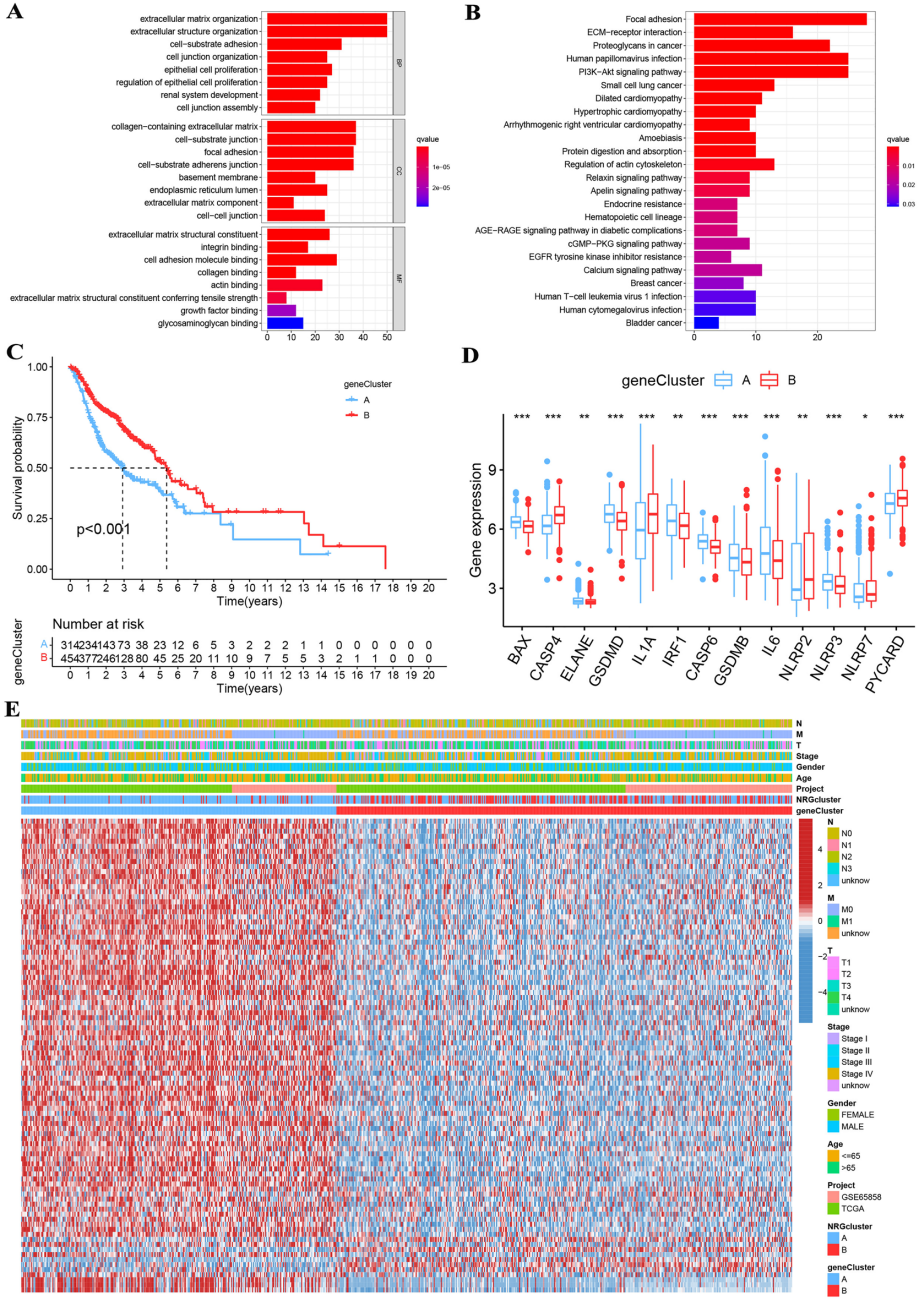
(**A**) These DEGs were abundant in biological processes, such as cell transfer and proliferation. (**B**) Enrichment of cancer-related pathways and immune systems was indicated by KEGG analysis. (**C**) Gene cluster B patients had the higher OS. (**D**) Important diversity was found in NRG expression of the two gene subtypes. (**E**) Gene expression and clinical features were significantly different.

The R package "limma" was applied to identify 251 DEGs associated with NRG subtypes, and functional enrichment analysis was performed to explore the potential biological behaviors. These DEGs were abundant in biological processes, such as cell transfer and proliferation (Fig. 4A and Tab S3). Enrichment of cancer-related pathways and immune systems was indicated by KEGG analysis, indicating that necroptosis plays an important role in the immune regulation of the TME (Fig. 4B and Tab S4). A total of 94 genes associated with OS time were selected by univariate Cox analysis (*p* < 0.05: Tab S5 and Fig S2A). Consensus clustering algorithms were applied to classify patients into two gene subtypes on the basis of prognostic genes to verify this regulatory mechanism (Fig S2B). Patients with gene cluster B had the highest OS, while patients with gene cluster A showed poor OS (*p* < 0.001; Fig. 4C). Important diversity was found in NRG expression of the two gene subtypes, consistent with our expected results (Fig. 4D). Gene expression and clinical features were significantly different, as demonstrated by comparing the clinical features of different genetic subtypes (Fig. 4E).

### Development and validation of prognostic model

**Figure 5:**
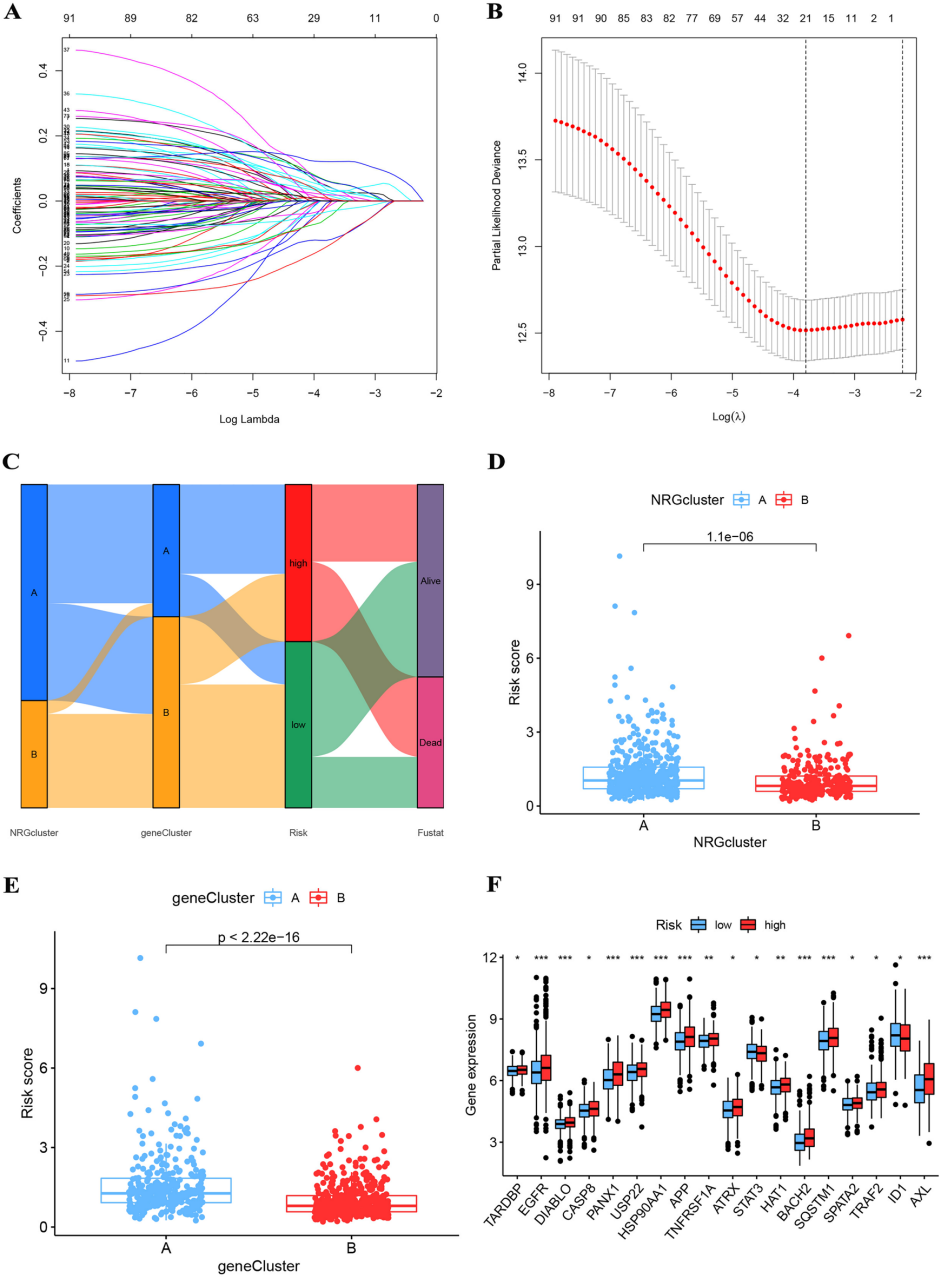
(**A**, **B**) The LASSO regression algorithm to screen candidate prognostic 21 DEGs. (**C**) The relationship between NRG cluster, gene cluster, risk groups, and survival status was visualized in a Sankey diagram. (**D**, **E**) The risk scores were significantly higher in NRG and gene cluster A. (**F**) Immune checkpoint genes were expressed at higher levels in the high-risk group.

To begin, we implemented a random division of the patient cohort into training and testing sets at a proportion of 7:3. Subsequently, to identify an optimal prognostic model, we subjected a total of 94 DEGs with prognostic potential to both LASSO and multivariate Cox analyses. In order to determine the most appropriate prognostic model, we applied a LASSO analysis, which yielded a set of 21 DEGs (Fig. 5A, B). We then conducted a multivariate Cox analysis to establish a predictive model that consisted of 15 DEGs (Fig S2C, and Tab S6). The risk model was developed as follows: risk score = (0.152 * THBS1) + (− 0.208 * LAMC2) + (0.223 * EFNB2) + (0.238 * P4HA1) + (− 0.118 * APCDD1) + (0.112 * F2RL1) + (− 0.274 * FERMT2) + (− 0.245 * PTPRZ1) + (0.127 * STC2) + (0.100 * FST) + (0.124 * MME) + (− 0.208 * ACTA2) + (− 0.083 * DEFB1) + (0.085 * SPP1) + (0.071 * ACTC1). A Sankey diagram was employed to visually demonstrate the interrelationship among NRG cluster, gene cluster, risk groups, and survival status (Fig. 5C). In addition, the risk scores were significantly higher in NRG and gene cluster A (Fig. 5D, E). Furthermore, immune checkpoint genes were expressed at higher levels in the high-risk group (Fig. 5F).

**Figure 6:**
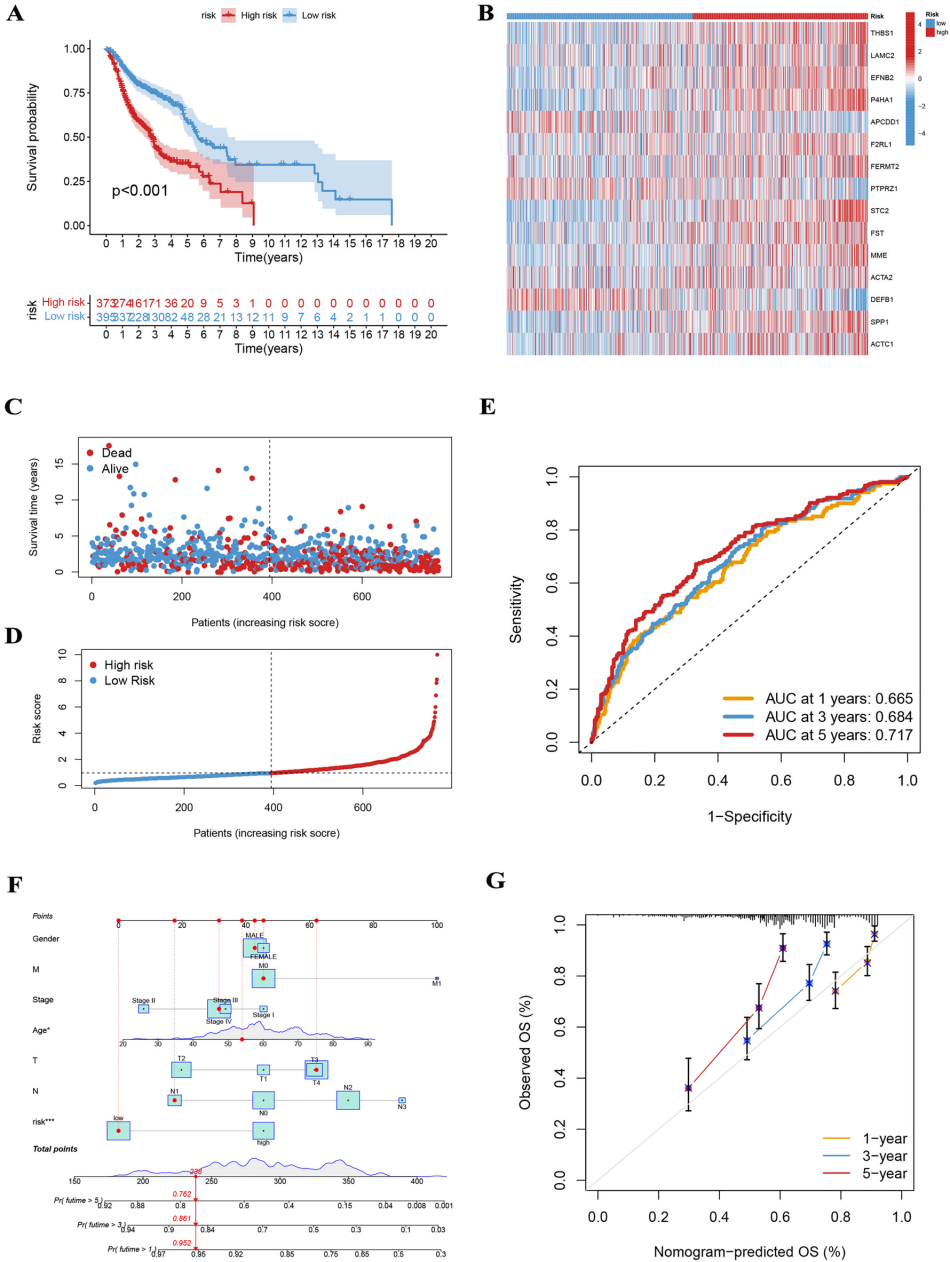
(**A**–**D**) The Kaplan–Meier analysis, expression profiles, survival status, and risk scores in the entire cohort. (**E**) The 5-year AUC value was 0.717 for the entire dataset. (**F**) The nomogram incorporating the signature and clinical characteristics was reliable and sensitive for predicting survival. (**G**) Calibration curves for OS.

Figure 6A–D provide comprehensive visualization of Kaplan–Meier analysis, expression profiles, survival status, and risk scores, collectively supporting the notion of a better prognosis for patients assigned to the low-risk group. Furthermore, the model exhibited a high degree of sensitivity and specificity for survival prediction, as evidenced in ROC curves, wherein the 5-year AUC value was 0.717 for the entire dataset (Fig. 6E). The reliability of the model was underlined by the results of our training and testing analyses, as represented in Figs S3 and S4, respectively. Additionally, the incorporation of the signature and clinical characteristics into the nomogram revealed it to be robust and sensitive for predicting survival, as depicted in Fig. 6F, G.

### Evaluation of immune landscape

**Figure 7:**
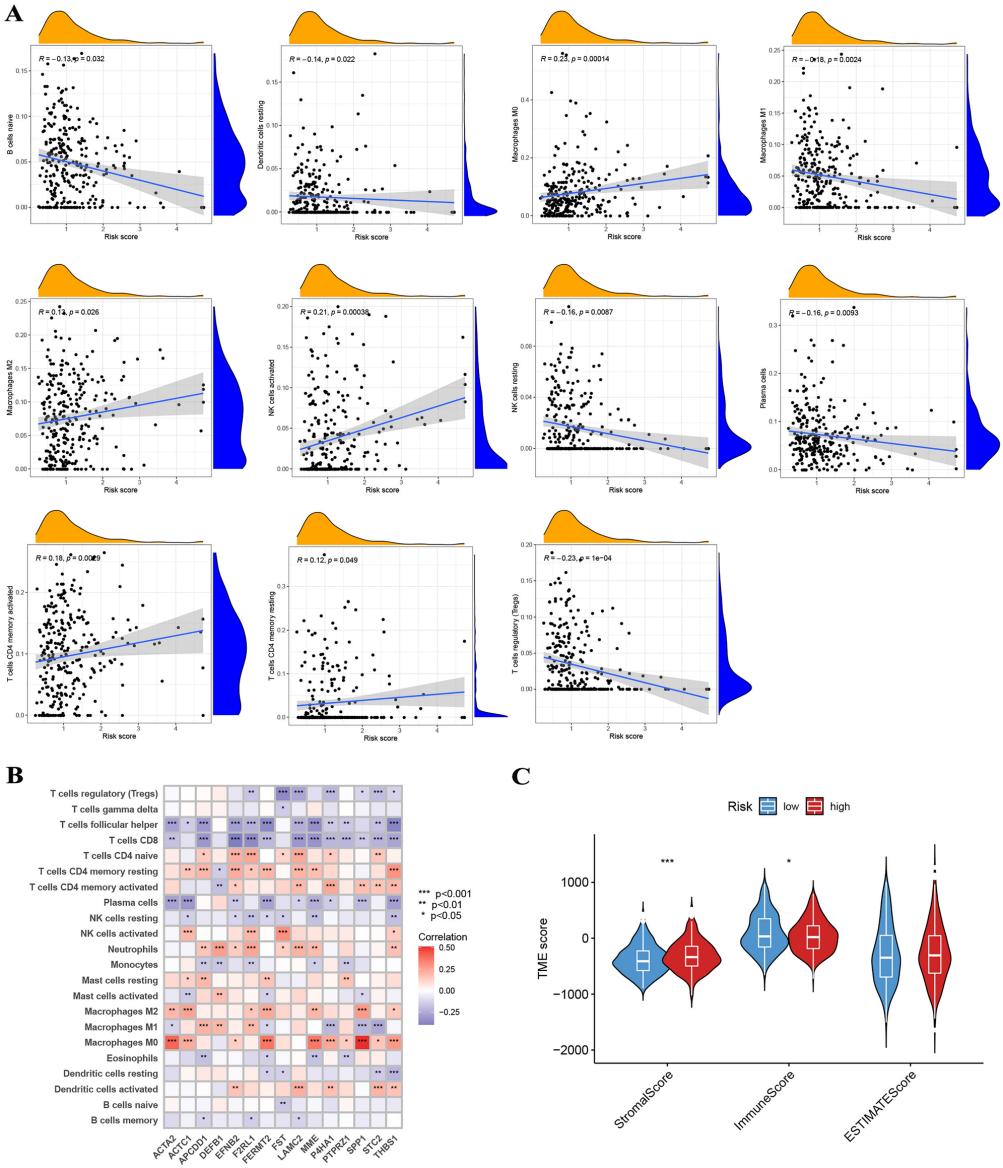
(**A**) The risk score exhibited a negative correlation with regulatory T cells, M1 macrophages, naive B cells, plasma cells, resting dendritic cells, and resting NK cells, while exhibiting a positive correlation with M0 macrophages, M2 macrophages, activated NK cells, memory CD4^+^ T cells, and resting memory CD4^+^ T cells. (**B**) The 15 genes are substantially linked with the majority of immune cells. (**C**) Higher Stromal scores and lower Immune scores in the high-risk group, with no significant difference in ESTIMATE scores.

To establish the relationship between the risk score and the quantity of immune cells present, the CIBERSORT algorithm was employed in our analysis. The scatter diagrams revealed that the risk score exhibited a negative correlation with regulatory T cells, M1 macrophages, naive B cells, plasma cells, resting dendritic cells, and resting NK cells, while exhibiting a positive correlation with M0 macrophages, M2 macrophages, activated NK cells, memory CD4^+^ T cells, and resting memory CD4^+^ T cells (Fig. 7A). Additionally, our evaluation of the connection between the number of immune cells present and the 15 genes integrated into the model indicated a significant linkage between most immune cells and the 15 gene signatures (Fig. 7B). TME score results showed higher Stromal scores and lower Immune scores in the high-risk group, with no significant difference in ESTIMATE scores (Fig. 7C).

### Analysis of mutation and CSC index

**Figure 8:**
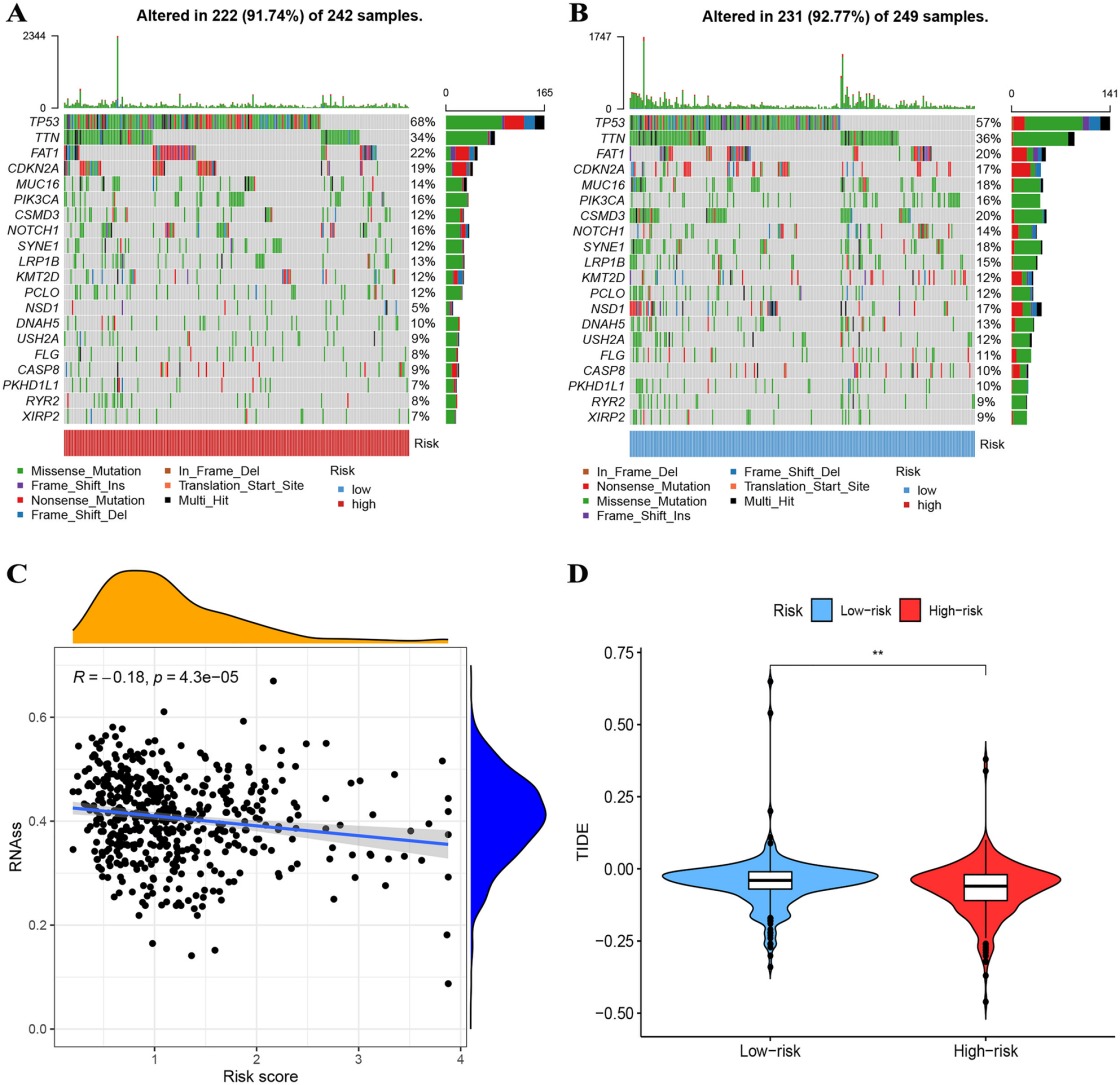
(**A**, **B**) The top 10 mutant genes were TP53, TTN, FAT1, CDKN2A, MUC16, PIK3CA, CSMD3, NOTCH1, and SYNE1. (**C**) HNSCC with lower risk scores exhibit more distinct stem cell traits and are less differentiated. (**D**) TIDE scores were lower in the high-risk group, indicating greater responsiveness to immunotherapy.

We identified the top 10 mutant genes in our cohort, which included TP53, TTN, FAT1, CDKN2A, MUC16, PIK3CA, CSMD3, NOTCH1, and SYNE1 based on the frequency of their occurrence (Fig. 8A, B). Our results indicated that the high-risk group exhibited greater frequencies of TP53, TTN, and FAT1 mutations compared to the low-risk group. On the other hand, while TTN and MUC16 mutations were highly prevalent in the low-risk group, their frequencies were reversed in the high-risk group. This observation underscores the importance of genetic variability in different risk groups and suggests that particular mutations may be more strongly associated with a higher cancer risk in the context of HNSCC. Furthermore, we also investigated the relationship between the risk score and the CSC index. Our data revealed an inverse correlation between risk score and CSC index (R = − 0.18, *p* < 0.001), indicating that HNSCC cells with lower risk scores exhibit more distinct stem cell traits and are less differentiated (Fig. 8C). This finding suggests that the risk score may potentially be utilized as a biomarker to assess the degree of stemness in HNSCC cells and thus predict the progression of HNSCC.

### Prediction of immunotherapy and identification of drugs

In the present study, we investigated the TIDE scores in relation to high-risk groups and found that TIDE scores were lower in the high-risk group, indicating greater responsiveness to immunotherapy (Fig. 8D). Moreover, by analyzing the IC50 of 29 commonly used antitumor drugs, we observed a significant statistical difference in the sensitivity of patients from different groups to these drugs (Fig S5). Our findings suggest that a more personalized approach to cancer treatment is necessary, where patient-specific factors are considered when determining the optimal treatment strategy. These results highlight the potential for improved therapeutic outcomes by utilizing a precision medicine approach in cancer therapy.

### Validation of prognostic DEGs

**Figure 9:**
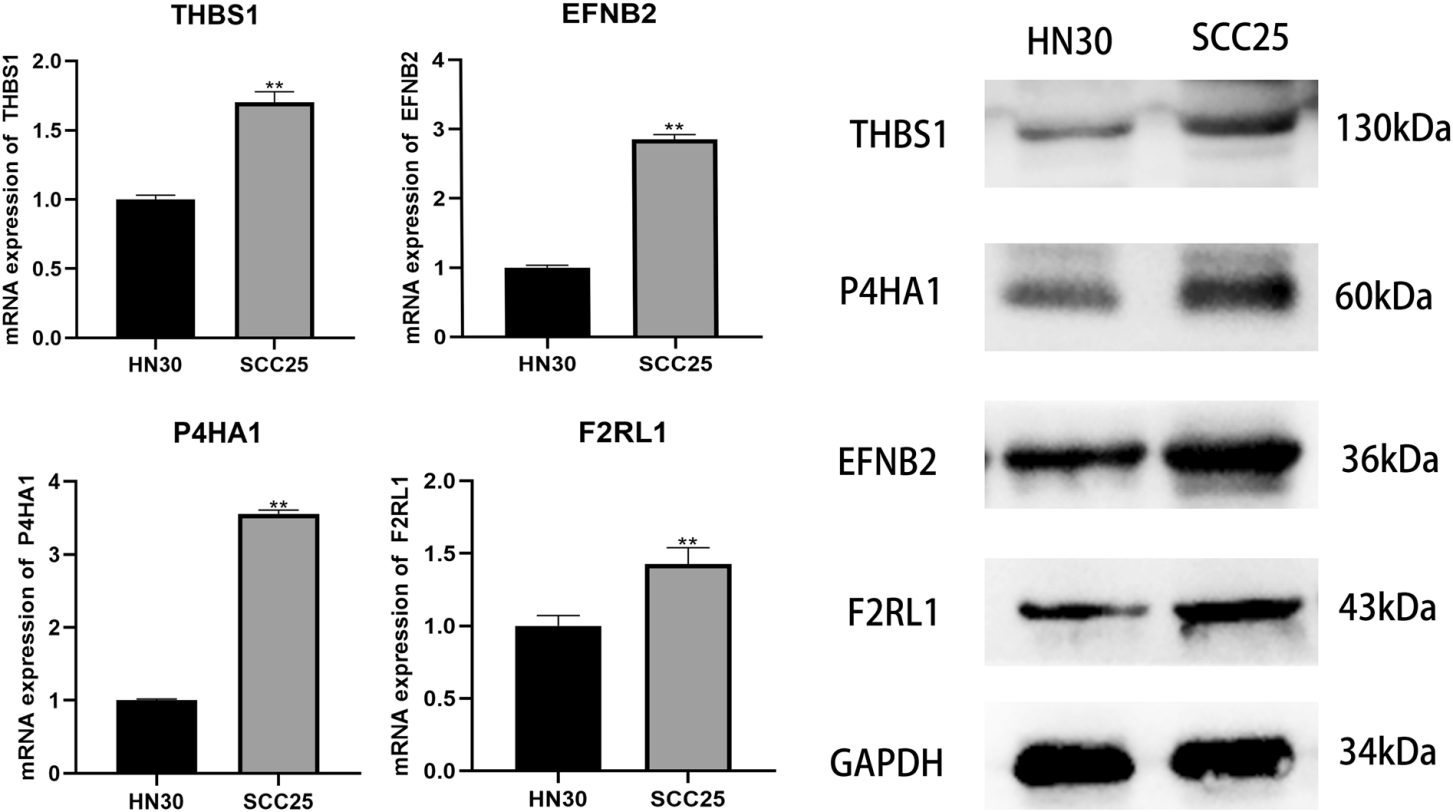
THBS1, EFNB2, P4HA1, and F2RL1 had higher levels of mRNA and protein in SCC25 cells compared to WSU-HN30 cells.

**Figure 10:**
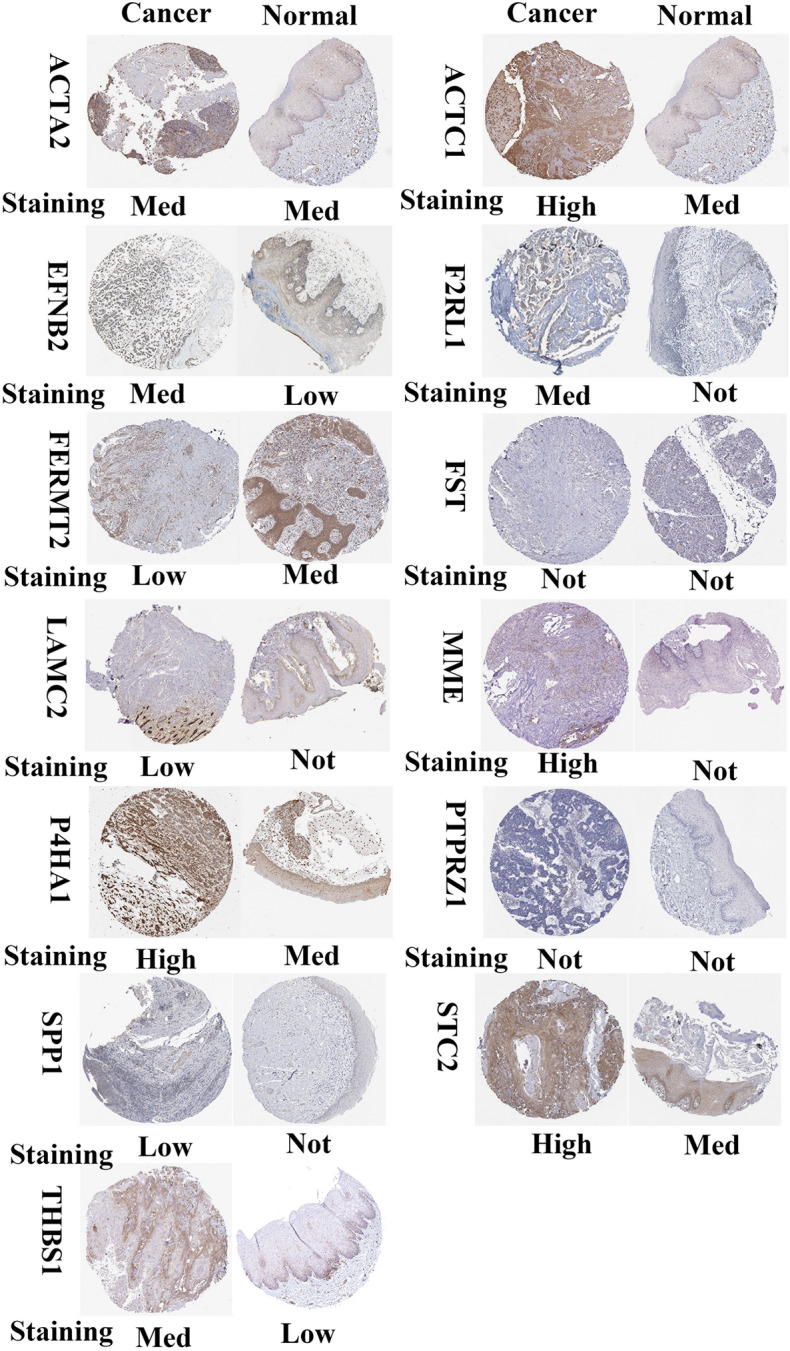
Several proteins including ACTC1, EFNB2, F2RL1, MME, P4HA1, SPP1, STC2, THBS1, and LAMC2 were found to be significantly overexpressed in tumor tissues of HNSCC compared to normal tissues. Conversely, FERMT2 exhibited a significant downregulation in HNSCC tumor tissues.

In this study, we aimed to further validate our findings regarding prognostic DEGs in HNSCC. To achieve this, qRT-PCR analysis and western blotting analysis were employed to confirm the expression levels of mRNA and protein in four DEGs (THBS1, EFNB2, P4HA1, and F2RL1) in WSU-HN30 and SCC25 cells. The findings showed that THBS1, EFNB2, P4HA1, and F2RL1 had higher levels of mRNA and protein expression in SCC25 cells compared to WSU-HN30 cells (Fig. 9). Moreover, the current study aimed to analyze the protein expression levels of tumor tissues and normal tissues in HNSCC using the HPA database. It is noteworthy that several proteins, including ACTC1, EFNB2, F2RL1, MME, P4HA1, SPP1, STC2, THBS1, and LAMC2, were found to be significantly overexpressed in the tumor tissues of HNSCC compared to normal tissues. Conversely, FERMT2 exhibited a significant downregulation in HNSCC tumor tissues (Fig. 10). This observation further validates the reliability and stability of our approach to identifying and analyzing prognostic DEGs in HNSCC.

## Discussion

Necroptosis plays a critical role in innate immunity and has anti-cancer effects^34,35^. The comprehensive impact and regulatory characteristics of TME infiltration mediated by multiple NRGs remain poorly understood^36^. However, the majority of research has focused on individual NRGs or TME cells. Our present study reveals significant dysregulation of NRGs in HNSCC at both the transcriptional and genomic levels, pointing towards widespread abnormalities in NRGs in this cancer type.

ZZW-115, a synthesized NUPR1 inhibitor, has been found to suppress xenograft pancreatic tumor growth by inducing mitochondrial metabolism rupture and necrosis^37^. Smac mimetics have been shown to induce necrosis in caspase-8-deficient colorectal cancer cells, inhibiting tumor growth and proliferation in mouse colorectal cancer models^38^. Shikonin has been demonstrated to inhibit osteosarcoma progression in vivo by promoting osteonecrosis activity^39^. Polyinosinic acid has been reported to induce necrosis in cervical cancer cells through RIPK3 expression, suggesting that the ubiquitin–proteasome system may regulate RIPK3-dependent necroptosis and that targeting this pathway with proteasome inhibitors may be a potential strategy for anti-tumor drug development^40^. Birinapant, a Smac mimetic, in combination with the caspase-8 inhibitor, Emricasan, has been found to induce necrosis in myeloid leukemia cells and may be a promising treatment for acute myeloid leukemia^41^. However, necrosis of tumor cells has also been implicated in promoting cancer cell dissemination and metastasis. Necroptosis biomarkers were found around necrotic foci in both mouse and human breast cancer tissues, and enhanced necroptosis activity was associated with increased breast cancer progression and metastasis^42^. Apoptosis induction has been shown to enhance invasion and migration of HNSCC and strongly correlate with HNSCC patient survival^43^. While some studies demonstrate the anti-tumor functions of necroptosis, growing evidence implies that necroptosis may promote tumor progression and metastasis, indicating that the role of the necroptosis pathway is likely dependent on cancer type. Therefore, it is crucial to identify specific necroptosis markers, investigate the molecular mechanisms, physiological and pathological functions of necrosis, and understand its interactions with other cell death mechanisms and the immune system.

Based on a set of 67 NRGs, we have identified two distinct molecular subtypes in HNSCC. Patients with subtype A had poorer overall survival, and differences in TME were observed between the two subtypes. Using the differentially expressed genes between the two necroptosis subtypes, we identified two genetic subtypes, suggesting that NRGs could serve as a predictor for clinical prognosis and immunotherapy response in HNSCC. Additionally, we established a reliable NRG score as a prognostic indicator, with immune activation and inhibition-driven necroptosis patterns displaying higher and lower NRG scores, respectively. Patients' TME, immune checkpoint expression, CSC index, prognosis, mutation, and treatment susceptibility varied significantly among different groups. Lastly, we developed a quantitative nomogram by combining the risk score and tumor stage, which greatly improved performance and facilitated the use of the risk score. This signature can aid in classifying the prognosis of HNSCC patients, contribute to a deeper understanding of the molecular mechanisms of HNSCC, and suggest new concepts for targeted treatments.

HNSCC patients have a poor prognosis following standard treatment, owing to the elevated number of checkpoints, tumor-infiltrating lymphocytes, and tumor neoantigens. Although recent immunotherapy advancements have been made, HNSCC patients still exhibit significant heterogeneity, underscoring the crucial role of TME in HNSCC tumor development. The main components of the TME are immune cells, including macrophages, lymphocytes, and granulocytes, which participate in various immune responses and activities, including the inflammatory response that contributes to cancer survival^44^. Evidence further supports that TME plays a vital role in cancer initiation, progression, and resistance to therapy^45,46^. In the current study, immunological inhibition-driven necroptosis (subtype A) was related to a higher risk score, while immune activation-driven necroptosis (subtype B) was associated with a lower risk score. The two molecular subtypes and different risk scores considerably varied in TME characteristics and the relative abundance of 22 immune cells.

In the management of early-stage HNSCC, surgical excision and radiotherapy are among the primary interventions employed. Similarly, advanced HNSCC is most effectively treated with a multimodal therapeutic approach involving surgery, followed by either postoperative chemoradiotherapy or upfront chemoradiation, with the consideration of surgical salvage if feasible^47^. Despite the potential benefits, induction chemotherapy has seen limited use, and its utility remains controversial, with seemingly inconsistent results in previous studies^48^. In this study, our findings revealed substantial differences in the IC50 values among different risk groups for a range of commonly used chemotherapeutic agents, namely Cisplatin, Docetaxel, Gemcitabine, Vinorelbine, and Paclitaxel. This observation signifies a potential avenue for tailoring treatment strategies to individual patients, emphasizing the importance of personalized medicine.

Delta gamma T lymphocytes have been found to accurately identify and eliminate HNSCC cells by inhibiting tumor growth through several mechanisms^49,50^. Subtype B and low-risk group patients with a better prognosis demonstrated increased infiltration of activated memory CD4^+^ and CD8^+^ T cells, as well as gamma delta T cells, suggesting that these cells may positively contribute to the progression of HNSCC. Conversely, Treg infiltration, which inhibits the anti-cancer response of the immune system, has been associated with a poor prognosis, which our findings are consistent with, as subtype B and high-risk group patients had greater Treg infiltration in the TME^51^. Additionally, B cells have been found to contribute to the immunological response^52,53^.

Previous research has demonstrated the significance of B-cell enrichment in predicting prolonged survival in soft tissue sarcoma patients treated with PD-1 inhibition, with B-cell enrichment serving as the most critical prognostic predictor^54^. Helmink et al.^53^ found that patients who responded to immune checkpoint blockade had significantly upregulated levels of B cell-associated genes such as MZB1, JCHAIN, and IGLL5. Additionally, tumor-infiltrating B lymphocytes have been associated with a favorable prognosis in HNSCC patients, with substantial B cell infiltration in metastatic HNSCC patients being linked to prolonged overall survival and a lower chance of disease recurrence ^55,56^. These findings underscore the significance of B lymphocytes as a target for cancer immunotherapy, proposing a promising new option for cancer treatment. In the present study, we observed no significant difference in memory B cell infiltration between the two NRG score groups and subtypes. However, subtype B exhibited reduced naive B cell levels and a higher NRG score, which was associated with poorer overall survival. Consistent with prior research, our findings suggest that B cell infiltration may inhibit HNSCC tumor growth^55^.

M1 and M2 macrophages are the two primary phenotypes of tumor-associated macrophages, with M1 macrophages inhibiting cancer progression and generating type I pro-inflammatory cytokines that exhibit antitumor properties^57^. Conversely, M2 macrophages facilitate matrix remodeling and immune suppression while promoting cancer formation^57^. Previous investigations have demonstrated that M2 macrophages are associated with epithelial-mesenchymal transition and can promote metastasis in HNSCC patients^58^. Furthermore, patients with high cancer stromal M1:M2 macrophage density ratios exhibit improved survival rates, whereas increased cancer stromal M2 macrophage density has been linked to lower cancer-specific survival. Consistent with prior investigations, we observed that the low-risk group with a favorable prognosis had higher M1 macrophage infiltration, while the high-risk group with a less favorable prognosis had increased M2 macrophage infiltration. These findings suggest that M1 and M2 macrophages may play a crucial role in HNSCC progression and prognosis, highlighting the potential use of macrophage-targeted therapies in the treatment of this condition.

There are several limitations in the present study that should be acknowledged. First, the investigation solely relied on data from the TCGA and GEO databases, which may have introduced inherent bias in case selection and influenced the resultant outcomes. To validate the current findings, further in vivo and in vitro experimental research as well as large-scale, prospective studies are warranted. Additionally, the paucity of data concerning several crucial clinical variables across most of the analyzed datasets may have potentially influenced immune responses and necroptosis conditions.

## Conclusion

Our comprehensive inquiry into NRGs indicated their impact on the TME, clinical manifestations, and outlook. Subsequently, we discerned the therapeutic imperative of NRGs in both immunotherapy and commonly prescribed antineoplastic agents. These discoveries underscored the pivotal clinical relevance of NRGs, offering a pioneering perspective for guiding immunotherapy and conventional antitumor treatment modalities for patients afflicted with HNSCC.

### Supplementary Information

Supplementary Legends.

Supplementary Figure S1.

Supplementary Figure S2.

Supplementary Figure S3.

Supplementary Figure S4.

Supplementary Figure S5.

Supplementary Legends.

Supplementary Tables.

## Data Availability

The datasets used and/or analysed during the current study are available from the corresponding author on reasonable request.
