# Supplementary material for: Revealing the potential of necroptosis-related genes in prognosis, immune characteristics, and treatment strategies for head and neck squamous cell carcinoma
Source: Sci Rep. 2023 Nov 21;13:20382. doi: 10.1038/s41598-023-47096-7 (PMC10663615; doi:10.1038/s41598-023-47096-7)
Supplement: Supplementary file 1 — Supplementary Legends. [file 41598_2023_47096_MOESM1_ESM.docx]

**Supplementary Figure S1.** Necroptosis scores did not differ significantly between early and advanced tumors.

**Supplementary Figure S2.** (A) 94 prognostic genes were selected by univariate Cox analysis (p <0.05). (B) Consensus matrix heatmap defining two gene clusters (k = 2). (C) 15 genes were applied in constructing the signature by multivariate Cox analysis.

**Supplementary Figure S3.** (A-D) The Kaplan-Meier analysis, expression profiles, survival status, and risk scores in the training cohort. (E) The ROC curves in the training cohort.

**Supplementary Figure S4.** (A-D) The Kaplan-Meier analysis, expression profiles, survival status, and risk scores in the testing cohort. (E) The ROC curves in the testing cohort.

**Supplementary Figure S5.** Recognition of anti-tumor drugs for the model.
