# Supplementary material for: Revealing the potential of necroptosis-related genes in prognosis, immune characteristics, and treatment strategies for head and neck squamous cell carcinoma
Source: Sci Rep. 2023 Nov 21;13:20382. doi: 10.1038/s41598-023-47096-7 (PMC10663615; doi:10.1038/s41598-023-47096-7)
Supplement: Supplementary file 7 — Supplementary Legends. [file 41598_2023_47096_MOESM7_ESM.docx]

**Supplementary Table S1.** The list of NRGs.

**Supplementary Table S2.** The GSVA analysis.

**Supplementary Table S3.** The of GO analysis.

**Supplementary Table S4.** The of KEGG analysis.

**Supplementary Table S5.** The details of univariate Cox analysis for DEGs.

**Supplementary Table S6.** The details of multivariate Cox analysis for candidate DEGs.
